# Identification of Weeds Based on Hyperspectral Imaging and Machine Learning

**DOI:** 10.3389/fpls.2020.611622

**Published:** 2021-01-25

**Authors:** Yanjie Li, Mahmoud Al-Sarayreh, Kenji Irie, Deborah Hackell, Graeme Bourdot, Marlon M. Reis, Kioumars Ghamkhar

**Affiliations:** ^1^AgResearch Ltd., Grasslands Research Centre, Palmerston North, New Zealand; ^2^Red Fern Solutions Ltd, Christchurch, New Zealand; ^3^AgResearch Ltd., Ruakura Research Centre, Hamilton, New Zealand; ^4^AgResearch Ltd., Christchurch, New Zealand

**Keywords:** hyperspectral imaging, weeds classification, superpixel, PLS-DA, multilayer perceptron

## Abstract

Weeds can be major environmental and economic burdens in New Zealand. Traditional methods of weed control including manual and chemical approaches can be time consuming and costly. Some chemical herbicides may have negative environmental and human health impacts. One of the proposed important steps for providing alternatives to these traditional approaches is the automated identification and mapping of weeds. We used hyperspectral imaging data and machine learning to explore the possibility of fast, accurate and automated discrimination of weeds in pastures where ryegrass and clovers are the sown species. Hyperspectral images from two grasses (*Setaria pumila* [yellow bristle grass] and *Stipa arundinacea* [wind grass]) and two broad leaf weed species (*Ranunculus acris* [giant buttercup] and *Cirsium arvense* [Californian thistle]) were acquired and pre-processed using the standard normal variate method. We trained three classification models, namely partial least squares-discriminant analysis, support vector machine, and Multilayer Perceptron (MLP) using whole plant averaged (Av) spectra and superpixels (Sp) averaged spectra from each weed sample. All three classification models showed repeatable identification of four weeds using both Av and Sp spectra with a range of overall accuracy of 70–100%. However, MLP based on the Sp method produced the most reliable and robust prediction result (89.1% accuracy). Four significant spectral regions were found as highly informative for characterizing the four weed species and could form the basis for a rapid and efficient methodology for identifying weeds in ryegrass/clover pastures.

## Introduction

Pastures based on perennial ryegrass (*Lolium perenne*) and white clover (*Trifolium repens*) are the main source of forage for animal production in New Zealand (McClearn et al., 2020). Weeds are a major economic constraint. Within the primary sector alone, weeds cost farmers NZ$50M in actual expenditure on chemical herbicides and labor (Bourdôt et al., 2007). Technologies that reduce these costs, and help minimize the use of synthetic herbicides, would improve the value of forage production (Bacco et al., 2018).

Recently, technologies such as hyperspectral imaging (HSI) systems are providing opportunities for rapid classification of plant species both in the laboratory and the field (Griffel et al., 2018; Liu and Zhang, 2018; Xu et al., 2018; Ferreira et al., 2019). The advantage of HSI is the provision of a combined spectroscopy and relationships between various chemical components and the absorption of spectra (Curran, 1989; Ebbers et al., 2002). The principle of HSI spectroscopy is based on molecular vibrations in the IR region (Youngentob et al., 2012). Therefore, absorbance at specific wavelengths, which might be related to specific chemical bands, can be used for different materials classification and quality determination (Vaiphasa et al., 2007; Schwanninger et al., 2011).

Many attempts have been made to use the visible light imaging or Red-Green-Blue (RGB) to identify weeds (Ahmad et al., 2018; Raja et al., 2020). However, shape, color and size, are limiting constraints of RGB imaging for the identification of species with similar phenotype (Wang et al., 2019). HSI can overcome these limitations by capturing spectral and spatial information simultaneously. It has a proven history of widespread use in materials discrimination and quality estimates including in meat science (Al-Sarayreh et al., 2018; Reis et al., 2018), forestry (Te et al., 2019), and land cover mapping (Jiang et al., 2017; Xu et al., 2018). Shorten et al. (2019) showed the potential of HSI for the measurement of a few components of forage quality. Other studies (Ahmad et al., 2019; Farooq et al., 2019b) indicate promising potentials for plant identification based on chemical signatures of different species.

Numerous analytical methods for HSI data classification have been reported (Lunga et al., 2014; Li et al., 2017; Audebert et al., 2019). Among these techniques, support vector machine (SVM; Yu et al., 2012; Peng et al., 2015) and partial least squares discriminant analysis (PLS-DA; Yang et al., 2015; Carreiro Soares et al., 2016; Walter et al., 2019) are considered as the most reliable techniques. This is specifically the case when limited training data are available (Melgani and Bruzzone, 2004; Chevallier et al., 2006).

Machine learning has been widely used for image classification (Chen et al., 2014). The Multilayer Perceptrons (MLP) methods have the advantage of handling a large number of training data (Golhani et al., 2018; Taneja, 2020). These methods could automatically learn features, while yielding comparable results on HSI classification process to other methods (Sutskever and Hinton, 2008).

Generally, the original HSI image contains the target (i.e., weeds) as well as the background and other components that could affect the labeling accuracy of the target species. To remove the background and obtain the region of interest (ROI), a segmentation strategy is required (Ren and Malik, 2003). Single or multistep thresholding algorithms are commonly used for obtaining the ROI and extracting the average spectrum of a sample (Sharma and Bhavya, 2020). Building prediction models based on the extracted spectra is commonly used for indoor applications (Mishra et al., 2017; Yuan et al., 2019). Spatial variation in spectra requires more attention in pixel-wise prediction and outdoor applications for achieving high prediction accuracy (Vaughn et al., 2016).

Image segmentation is a crucial step in analyzing and understanding the contents of an image. It can be used to extract a wide range of image features including spatial features and superpixel (Sp) segmentation is one of these segmentation methods (Ren and Malik, 2003). In this method the pixels are grouped into many small segments adhering to the target boundaries where each segment shares the same spectral and spatial features of a common target (Li and Chen, 2015). It provides a compact and uniform segmentation for the target and extracts the spatial spectra from the image (Fan et al., 2017).

Therefore, we hypothesize that there are unique spectral signatures in each weed species, which are detectable by HSI and modeling. To test our hypothesis, we used three common weeds and a proxy weed species in ryegrass paddocks of New Zealand (NZ). The three weed species were the annual winged thistle (*Carduus tenuiflorus* Curtis), the annual yellow bristle grass [*Setaria pumila* (Poir.) Roem. & Schult.] and the perennial giant buttercup (*Ranunculus acris* L.). Winged thistle is a problematic weed of drought-prone low-fertility sheep and beef cattle pastures while yellow bristle grass and giant buttercup are weeds of high-fertility dairy pastures (Bourdôt et al., 2003; Lamoureaux, 2014). The fourth species, wind grass [*Anemanthele lessoniana* (Steud.) Veldkamp], is a NZ native species. It was used as a proxy for Chilean Needle Grass [*Nassella nessiana* (Trin. & Rupr.)], which cannot legally be cultivated in NZ. This grass is currently limited in its geographical distribution in New Zealand but threatens vast tracts of low-fertility drought prone hill-country pasture land (Bourdôt et al., 2010, 2012). Proven true, this hypothesis will allow future development of database for spectral signatures of weeds, which is valuable to detect weeds independently of the type of fields they are found in (i.e., independently of the type of plant species that are surrounding the weeds).

## Materials and Methods

### Weed Sample Preparation

**FIGURE 1:**
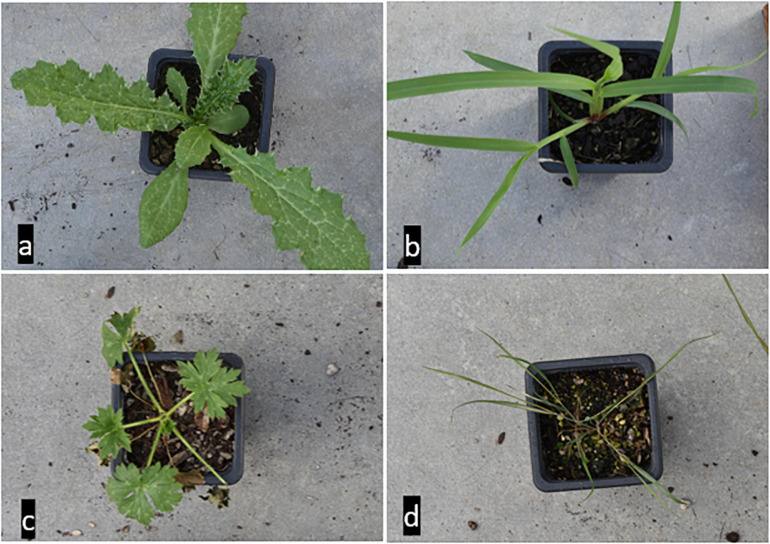
The four weed species **(a)**: Thistle (TT), **(b)**: Yellow bristle grass (YBG), **(c)**: Buttercup (BC), and **(d)**: wind grass (WG).

Four criteria were considered to choose the weed species, i.e., cover grass, broadleaf, perennial, and annual weeds. Selected weed species included three weeds of ryegrass pastures [thistle (TT), yellow bristle grass (YBG), and buttercup (BC)] and one proxy endemic species [wind grass (WG)] (Figure 1). Weed seeds were sourced from Margot Forde Germplasm Centre (MFGC), Palmerston North, NZ. For each weed species, 30 single seeds were planted in pots (one plant per pot) on 2 October 2018 at AgResearch Ruakura campus (Hamilton, New Zealand), The pots were standard 7 cm (7 × 7 × 8 cm) plastic pots placed on tables in the open-air greenhouse with a temperature between 18 and 25 degrees and watered as required (2–3 times a week). The standard Daltons potting mix soiled was used (40% bark fiber, 20% C.A.N Fines A grade, 20% Coco fiber classic, 20% pumice 7 mm plus fertilizer containing lime, permawet, osmocote, microplus (Te, Mg, and Fe), Gypsum, dolomite, and coated ExteNd). After 3 weeks, all plants were transferred to the lab for HSI scanning.

### Hyperspectral Imaging

A line scan HSI spectrograph system (Extended VNIR, Headwall Photonics, Fitchburg, MA, United States), with a 320 × 240 pixels camera was used for the HSI data collection. This system covered the range of 550–1,700 nm spectra with 5 nm spectral resolution and 235 wavelengths from the visible and NIR range of the electromagnetic spectrum. A halogen lamp light source (JCR 21V 150W/AL Japan 2DB) was set up on one side of the camera’s lens, at 30^*o*^ from the vertical plane as the illumination system. The light power was adjusted using a white reference tile (Labsphere Inc., North Sutton, NH, United States) where the highest intensity detected in the white reference tile (Labsphere Inc., North Sutton, NH, United States) was set as 85% of the saturation of the detector to prevent areas where the sample may saturate the detector. The distance between the plant sample and the camera was adjusted to 25 cm and the plants were placed directly below the HSI system with the camera exposure time set on 25 ms. The translation speed of the linear stage was set to 11.1 mm/s. The white reference image was captured by placing a white tile under the hyperspectral camera. Dark reference images were acquired with the lens cap on the hyperspectral camera.

Single weed-pots were placed on the linear stage to capture the hyperspectral images when they pass under the camera. Considering that each pot was scanned individually, the presence of shadows was not a major issue. For cases where the scanning is performed in the field, it is possible to use a different illumination system to reduce the presence of shadows (Bateman et al., 2020).

**FIGURE 2:**
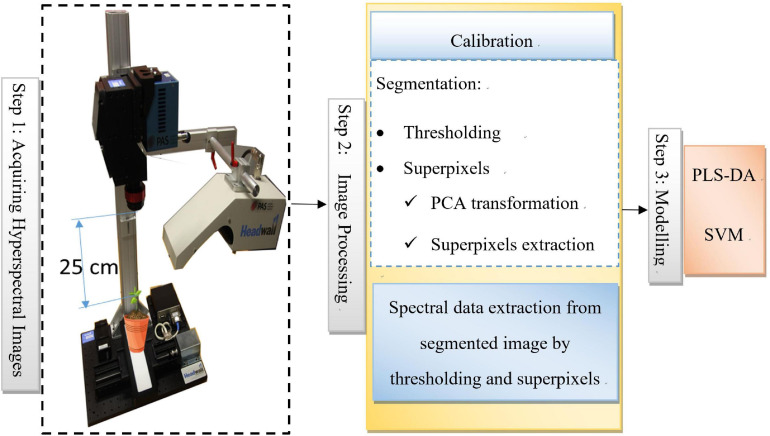
Proposed workflow for identification of Weeds.

Three steps of the workflow for identification of weeds are shown in Figure 2. These three steps are:

**Step 1: Acquiring hyperspectral images:**

Hyperspectral images were captured for 30 samples of each weed. Four types of weeds samples were present so in total, 120 hyperspectral images of weeds have been captured.

**Step 2: Image Processing**

Captured hyperspectral images were go through a series of steps to process them for extracting the spectral data for modeling.

**Step 3: Modeling**

Spectral data extracted from the image processing step pre-processed and then it was used as an input for model development.

### Image Processing

This was the second step of the workflow where ROI extracted by employing segmentation on calibrated hyperspectral images.

#### Calibration

Each captured hyperspectral image was calibrated, using dark reference (D), and white reference image (W). Hyperspectral images raw intensity values were used to calculate reflectance by using Eq. 1.

(1) $$R_{c}=\frac{X_{r\,a\,w}-X_{D}}{X_{w}-X_{D}}$$

where *R*_*c*_ is the absolute reflectance, *X*_*raw*_ stands for the intensity value of sample weeds scanned, *X*_*w*_ symbolizes the intensity value of captured white reference and *X*_*D*_ represents the intensity of the dark reference.

#### Segmentation

The aim of segmentation was to extract plant ROI by segregating the background (i.e., soil, stones, etc.) from the vegetation (i.e., leaves of different weeds). Custom code was generated in-house using R for thresholding segmentation and superpixel segmentation. Details of this are provided below.

**FIGURE 3:**
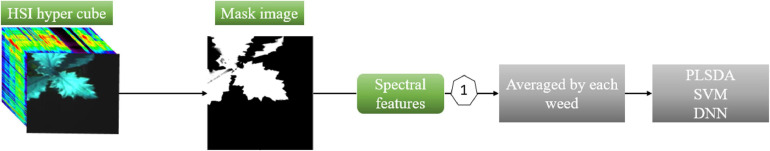
Thresholding segmentation to extract spectral features.

**FIGURE 4:**
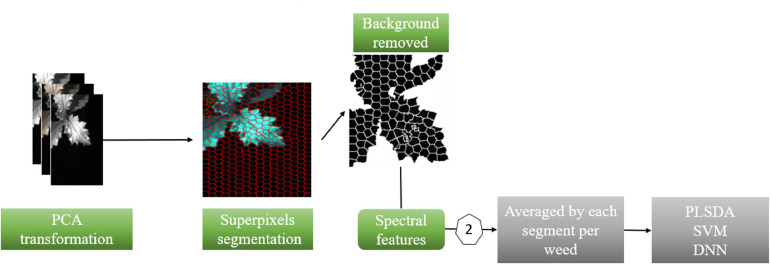
Superpixel segmentation to extract spatial-spectral features.

(a) Thresholding segmentation: A thresholding algorithm was developed by applying threshold value of 0.19 at 950 nm wavelength. This generated a mask which was then multiplied with the original HSI image to create an image of vegetation material only (Figure 3). The spectral data extracted after the thresholding segmentation was averaged and we named these averaged spectra as “Av” spectra which is the spectra for each plant with 235 components corresponding to 235 wavelengths. To obtain the Av, the mean spectrum of 120 segmented HSI images (one HSI image for each potted plant) was calculated on weed leaves resulting in the collection of 120 samples, which were used for training (96 samples), and validating (24 samples) the PLS-DA, SVM, and MLP models.

(b) Superpixel segmentation (Figure 4): The simple linear iterative clustering (SLIC) algorithm (Achanta et al., 2012) was used to divide the plant image into non-overlapping patches Sp (superpixels). This was achieved by taking the similarity in spectral and spatial domains into account when grouping pixels into clusters. Principal components analysis (PCA) was used to transform the original HSI image (where each pixel contained 235 wavelengths) into three channels (each pixel contained 3 principal components). In total, 120 PCA images were created. This was followed by the segmentation of each PCA image into 400 patches using SLIC algorithm. The patches that contained leaves were extracted and the mean spectrum of each valid patch was extracted from the HSI image and used as the spectral and spatial (Spectro-spatial) features of the weed leaves. All patches in each plant were marked with the same label. We denoted it as “Sp” averaged spectra which mean each plant has “n” “SP” spectra, each containing 235 elements corresponding to 235 wavelengths. “n” is the number of super-pixels for an individual plant. This number of clusters used in SLIC (i.e., 400 patches) was enough to avoid patches mixing regions from weed leaves and the background. While for this study the valid patches (which contained leaves) were separated manually, in a practical application a model may be used to separate between patches representing the plant leaves and the background.

### Modeling

Three commonly used discrimination models for object classification were used in this study. These were partial least squares-discriminant analysis (PLS-DA), SVM, and MLP.

#### Partial Least Squares-Discriminant Analysis

Partial least squares-discriminant analysis is a very popular linear classification method in chemometrics and is based on the PLS regression algorithm (Lee et al., 2018). In PLS-DA, the output (*y* variable) of PLS regression is transferred into a categorical structure as reference value and descriptor matrix *x* is used for discrimination analysis. It typically produces the lowest within-class variability and therefore maximum separation. The scores of latent variables (LVs) from the resulting PLS-DA model were used to identify groups of samples representing the four types/classes of weeds. The regression coefficients corresponding to those LVs with discrimination power between classes were then evaluated to identify the spectral regions potentially associated with discrimination of weeds (Wold et al., 2001; Barker and Rayens, 2003). This method has been widely used for identifying chemical traits and species classification in food and agriculture sciences (Bassbasi et al., 2014; Botelho et al., 2015; Lenhardt et al., 2015).

#### Support Vector Machine

Support vector machine analysis (Boser et al., 1992), is a powerful technique and ideal for data classification, especially for the high-dimensional data with a limited number of training samples (Tarabalka et al., 2010). This method was originally defined for binary classification and has been also extended to form a multi-class classification (Pérez-Cruz and Artés-Rodríguez, 2002). This extension allows for a broad application in hyperspectral image analysis (Fauvel et al., 2008; Pal and Foody, 2010) and remote sensing (Melgani and Bruzzone, 2004; Mountrakis et al., 2011).

#### Multilayer Perceptron

Multilayer perceptron is a powerful machine learning technique that can characterize the features of the samples and learn the appropriate classification features from the samples (Goodfellow et al., 2016). The MLP model is dependent on multiple sets of parameters, such as the number of hidden layers, regularization parameter, and activation epoch (Ramchoun et al., 2017). Activation function allows the introduction of non-linear function to the neural network. Activation epoch also prevents the MLP model from becoming a simple linear function with limited learning power. There are three main activation functions: hyperbolic tangent (Tanh; Kalman and Kwasny, 1992), rectifier (Xavier et al., 2011), and maxout (Goodfellow et al., 2013). The two types of regularization (L1 and L2) are useful functions in the MLP model to reduce the effect of overfitting.

### Multivariate Data Analysis

Data analysis was conducted in R software version 3.1.2 (R Core Team, 2017). The “mdatools” package (Kucheryavskiy, 2019) was applied for the PLS-DA model, and the “e1071” package (Meyer et al., 2019) for SVM model construction. The “h2o” package (Erin et al., 2019) was used for MLP modeling and variable selection.

Two types of data, i.e., (a) spectral data, i.e., “Av” and (b) combined spectral and spatial data “Sp” were used for training the model by using PLS-DA, SVM, and MLP methods. These included average of a leaf spectra for each weed and the average of each selected leaf patch of a weed species (Figures 3, 4). We chose 80% of each data set for model calibration and the remaining 20% for validation and elementary testing. Two assessments were used: (1) all the pixels from segmented plant; and (2) the averaged spectra of segmented plants. This generated two datasets: “all pixels dataset” and “average dataset.” Then each dataset was split into two sets. The “all pixels” generated “all pixels calibration dataset” and “all pixels validation dataset.” Similarly, the “averaged dataset” generated “averaged calibration dataset” and “averaged validation dataset.” Each calibration dataset was used to fit a model independently. Each model was then applied to the corresponding validation dataset. The assessment of predictions resulting from these two independent validation datasets resulted in two set of accuracies.

The pre-processing method SNV (Standard Normal Variate) was applied to the spectra before model calibration which has been shown to be a reliable pre-processing method on weed classification (Shirzadifar et al., 2018). The number of significant LVs for the PLS-DA, and the parameters of epsilon and cost for SVM models were determined using the leave-one-out cross-validation method (Sudheer et al., 2014; Vehtari et al., 2017).

#### Model Performance Metrics and Optimization

The parameters recall (R), precision (P), average accuracy (AA), and overall accuracy (OA) were used for PLS-DA, SVM, and MLP model performance. Four quantities from the performance of a classification process in the population of all instances were used to calculate R, P, AA, and OA: True positives (TP), false positives (FP), true negatives (TN), and false negatives (FN) using below the equations:

$$\text{R}=\frac{T\,P}{T\,P+F\,N}$$

$$\text{P}=\frac{\text{TP}}{T\,P+F\,P}$$

AA=TP+TN⁢T⁢P+T⁢N+F⁢P+F⁢N

$$\text{OA}=\frac{{\text{AA}}_{1}+{\text{AA}}_{1}+{\text{AA}}_{\ldots .}+{\text{AA}}_{n}}{n}$$

where n is the number of the classes [thistle (TT), yellow bristle grass (YBG), buttercup (BC), and wind grass (WG)]. To qualitatively evaluate the predictability power of models for weed classification, the t-SNE algorithm (Maaten and Hinton, 2008) was applied. The accuracy of a classification process was defined as the portion of true positives and true negatives in all instances.

##### Model optimization

**FIGURE 5:**
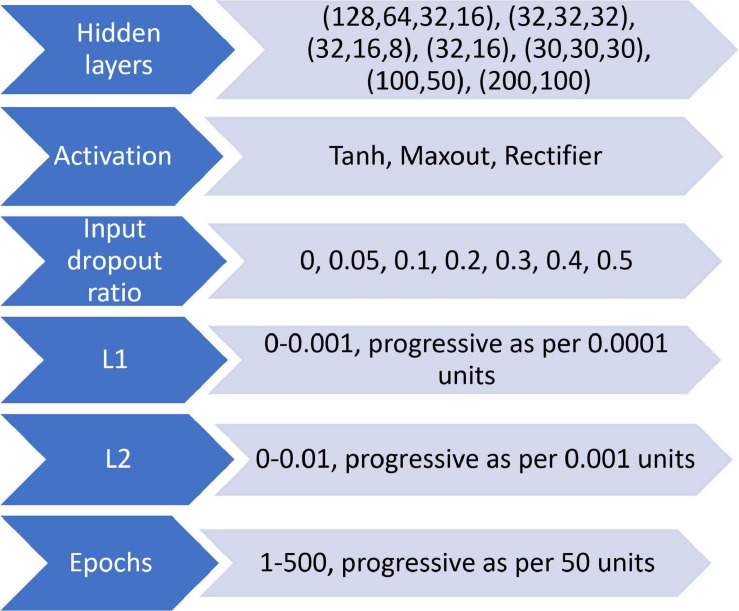
The loop structure for all MLP models.

To find the best parameters of the MLP model for Av and Sp data, a grid loop with different hidden layers, activation function, epochs and different l1 or l2 regularization parameters was set up (Figure 5). A five-fold cross-validation was used for fine-tuning these parameters. Models with the highest OA and the lowest loss values were chosen as the final model, and the feature weight from the final best performance model was used for feature evaluation.

## Results

### Mean Raw and Standard Normal Variate Spectra

**FIGURE 6:**
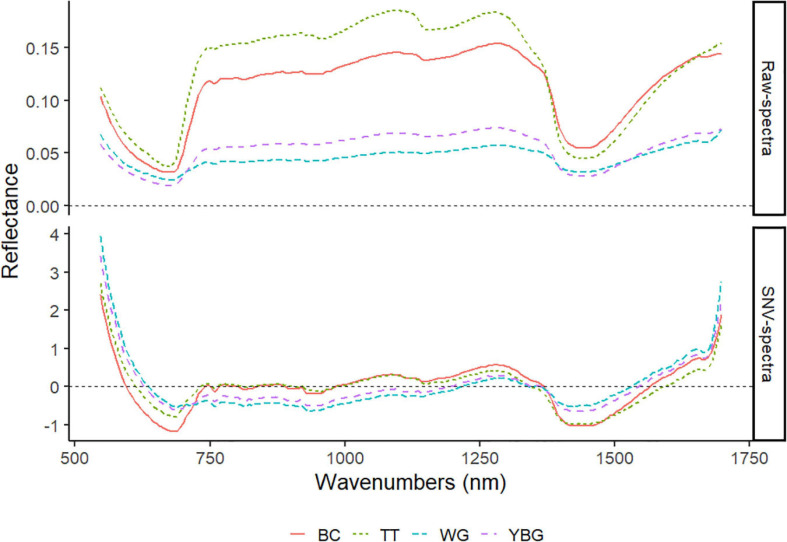
The raw and SNV VIS-NIR mean spectra of four weed species. TT: thistle, YBG: yellow bristle grass, BC: buttercup, and WG: wind grass.

The raw and SNV mean of 30 weeds for each species are plotted in Figure 6. While all weeds showed similar general patterns in both types of spectra, large variability between the four weeds was also observed. The SNV spectra highlights regions that can be used for discrimination between the four weed species. Three important regions were identified based on these two types of spectra: 550 to 700 nm, 1,000 to 1,200 nm, and 1,300 to 1,500 nm. These regions are the only regions in the spectra used in this study with detectable difference in reflectance value among the four weed species.

### Models Evaluation

The notations used for models developed with Av and Sp data using PLS-DA, SVM, and MLP are given below:

(a) Av_PLS-DA: PLS-DA model developed with Av data
(b) Sp_PLS-DA : PLS-DA model developed with Sp data
(c) Av_SVM: SVM model created with Av data
(d) Sp_SVM: SVM model created with Sp data
(e) Av_MLP: MLP model generated with Av data
(f) Sp_MLP: MLP model generated with Sp data

The optimal number of LVs was chosen as 10 for both Av and Sp_PLS-DA models based on cross validation. The optimal value of epsilon and cost for SVM model using Av and Sp data were 0 and 32, and 0 and 4, respectively. For the MLP model, Tanh activation with two hidden layers (32, 16) were selected for final application. The full-length spectra with 5% dropout was set as the input layer, and the four classification classes were set as the output layer. The validation set of both Av and Sp data were used to test the ability of our model for weed classification. The results of the modeling are presented in Table 1. Overall, the PLS-DA, SVM and MLP models yielded relatively high classification results based on both the Av and Sp data with an overall accuracy (OA) of 70–100%. MLP model yielded the highest recall (R), precision (P). Furthermore, average accuracy (AA) and OA with Av and Sp data were 1, 1, and 0.89, 0.90, respectively.

**TABLE 1:** Evaluation of the preformance of the proposed PLS-DA SVM, MLP models for weed recognition in the validation set, using four parameters: average accuracy (AA), overall accuracy (OA), recall (R), and precision (P), based on Av and Sp data.

		**Av data**				**Sp data**			
		***R* (%)**	***P* (%)**	**AA (%)**	**OA (%)**	***R* (%)**	***P* (%)**	**AA (%)**	**OA (%)**
PLS-DA	YBG	100	83	87	91	70	96	70	70
	BC	80	100			63	64		
	TT	100	100			72	88		
	WG	67	100			76	25		
SVM	YBG	100	100	92	92	92	90	84	86
	BC	80	80			73	81		
	TT	100	100			86	92		
	WG	89	89			86	61		
MLP	YBG	100	100	100	100	94	91	89	90
	BC	100	100			82	81		
	TT	100	100			90	95		
	WG	100	100			89	79		

*TT: thistle, YBG: yellow bristle grass, BC: buttercup, and WG: wind grass.*

**FIGURE 7:**
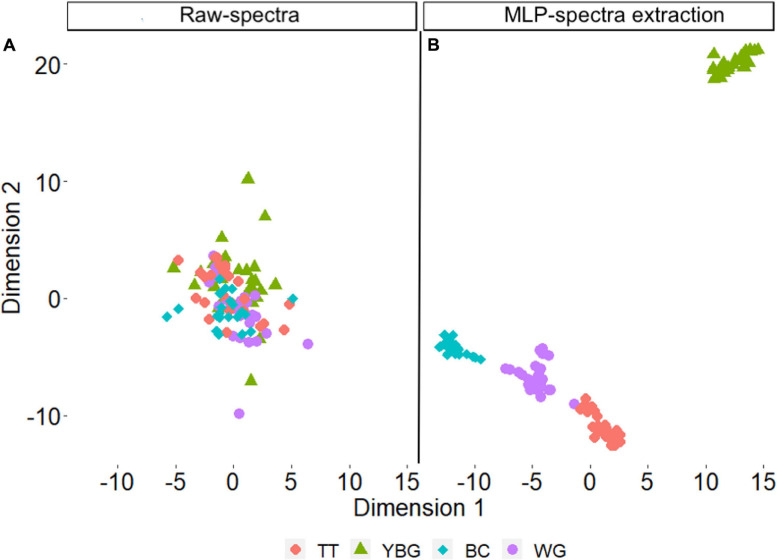
The prediction performance of Av_MLP using spectral data of four weed **(A)** raw avearage spectral data **(B)** features data that extracted from Av_MLP model hidden layer.

Multilayer Perceptron models were best performing models with Av and Sp data. The t-SNE algorithm was applied to the features that were extracted from Av_MLP model hidden layer and the raw Av spectral data for comparison. The results showed that the raw Av data did not discriminate the four species (Figure 7A). However, they were distinctly classified after the application of the MLP extraction model (Figure 7B).

### Performance of the Models on All Hyperspectral Pixels

**FIGURE 8:**
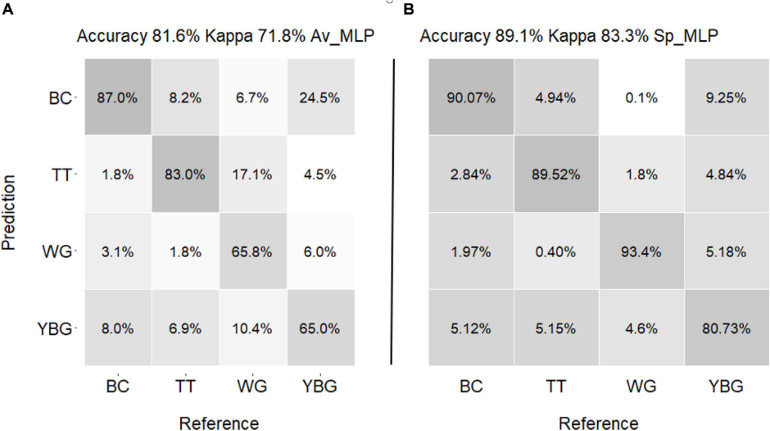
Confusion matrix of predicted weed species for validation set **(A)** by Av_MLP **(B)** by Sp_MLP. The numbers in the matrices are each weed prediction percentage. (TT: thistle, YBG: yellow bristle grass, BC: buttercup, and WG: wind grass).

The confusion matrix of the actual weeds and predicted weeds by Av_MLP and Sp_MLP models are shown in Figure 8. The Sp_MLP model produced higher prediction accuracy than the Av_MLP model. The prediction accuracy of Av_ MLP model (81.6%) using all pixels validation dataset was lower than the accuracy of using averaged validation dataset (100%; Table 1). The Sp_MLP and Av_MLP models yielded similar accuracy for the predication of all pixels with 89.1 and 81.6% accuracy, respectively. Av_MLP performed lower accuracies (65 and 65.8%) than the Sp_MLP model (80.7 and 93.4%) for the identification of YBG and windgrass. These results suggest that averaging all spectra across the weed species enhances the amount of information captured about the weeds. However, when spectra come from smaller regions (e.g., single pixel, from Sp) this ability is reduced.

**FIGURE 9:**
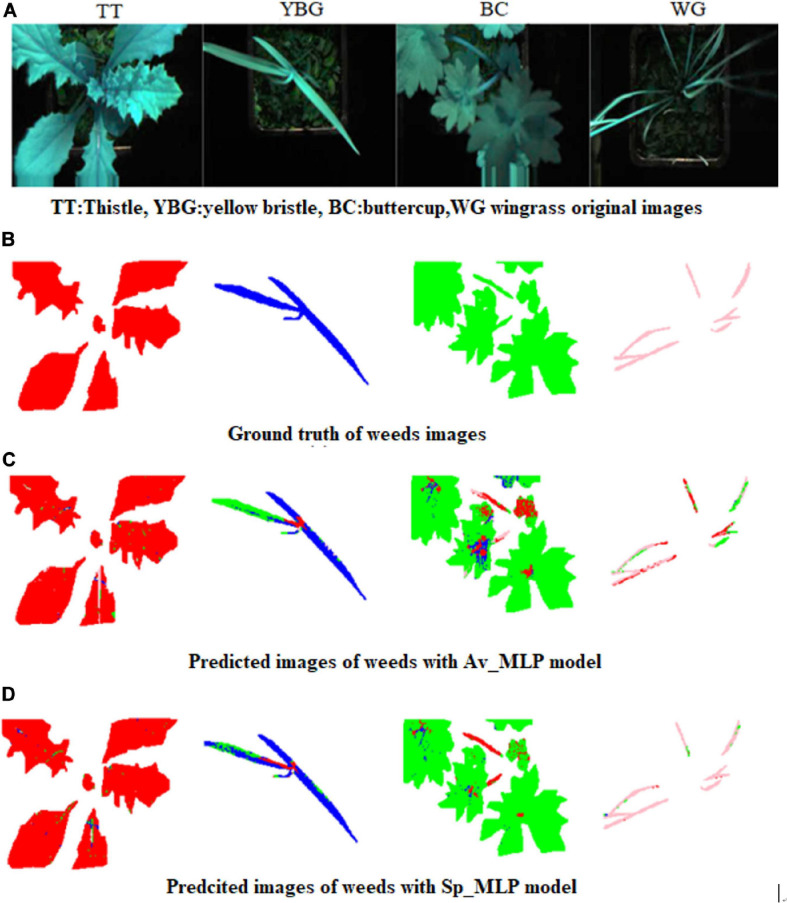
False color and predicted images of validation set for weeds **(A)** False color images of the weeds : TT: thistle, YBG: yellow bristle grass, BC: buttercup, and WG: wind grass **(B)** generated ground truth images **(C)** Predicted image with Av_ MLP model **(D)** Predicted image with Sp_MLP model. Note: Four prediction colors were used; Red: Thistle, Blue: YBG, Green: Buttercup, and Pink: Windgrass and a hard threshold was used to remove the background of the leaves. The different colors are representing different weeds, which means that some pixels in this image have been predicted as other plants, and not all pixels have been predicted correctly.

The predicted, ground truth and false color images of the weeds are presented in Figure 9. From Figures 9C,D we can conclude that Sp_MLP model showed better precision in overall weed recognition (i.e., for YBG, BC, and WG weeds) than the Av_MLP model. Furthermore, in the case of thistle, similar prediction was observed by Sp_MLP and Av_MLP models.

## Discussion

Targeted weed control could increase the speed and accuracy and reduce costs to farmers (Komi et al., 2007). The prerequisite of targeted weed control is a reliable weed identification system. Many techniques have been used in recent years to increase the accuracy and speed of weed identification mainly applying RGB imaging (Solahudin et al., 2018). HSI, however, has the advantage of identifying weeds based on their reflectance data, which is an indicator of the plant’s chemical composition (Farooq et al., 2019a). Using HSI will add value to the weed identification techniques based on RGB imaging, which is hinged on shape, size, and color discrimination. Wei et al. (2014) applied canonical discriminant analysis and the PLS-DA model to data from five wavelengths (672, 757, 897, 1,117, and 1,722 nm) to discriminate soil and five weed species from winter rape. They achieved this with a high accuracy of 90.91%, which is slightly higher than our result. The reason for this might be that they only discriminated the broad leave species, whereas we have very narrow-leaf species of grass as well. Broad leaf species are slightly simpler to identify than the narrow leaf species because of the fact of more uniform spectral data collection.

The MLP method has been widely used for classification in agricultural research (Golhani et al., 2018; Kiani et al., 2018). In our study, four important spectral regions (550–750, 995–1,005, 1,110–1,220, and 1,380–1,470 nm) have been identified by Sp_MLP model as “the best model” with high weed identification performance. In general, the 500–750 nm spectral region has been reported as important in vegetation discrimination (Cochrane, 2000; Smith and Blackshaw, 2003; Frels et al., 2018). Further, the region around 700 nm is known to be highly informative for vegetation discrimination due to its association with chlorophyll content (Gitelson and Merzlyak, 1997). The spectra in the ranges of 880–1,000,1,050–1,200, and 1,250–1,550 nm has been mostly associated with the third and second overtone of C-H stretching and second overtone of O-H stretching (Burns and Ciurczak, 2007; Schwanninger et al., 2011). Danson et al. (1992) described that the bands at 970, 1,200, and 1,450 nm wavelengths are water absorption bands.

All three identification models used in this study yielded high prediction accuracy both based on the Av and Sp selected spectra. However, the MLP model produced the highest accuracy, sensitivity and speciality compared to the other identification models both in the calibration and validation sets using the Av and Sp data. The t-SNE method has been recognized as a very powerful approach for data exploration and visualizing high-dimensional data (Maaten and Hinton, 2008). In this study, the first two dimensions of t-SNE extracted from original spectra and Av_MLP showed that the original spectra do not efficiently discriminate the four weed species. However, the discrimination power was improved by the Av_MLP model, with four weeds identified and discriminated.

The Av spectral models also distinctly identified the four weed species. This is likely due to chemical composition of each species (Vengris et al., 1953). However, the data could not be simply averaged for model calibration when the model was used for classification across all pixels obtained from hyperspectral images. According to (Fang et al., 2015) the shape of the hyperspectral images should be assessed based on the different structures of HSI specifically if the heterogeneous spatial area is large. Averaging each selected Sp area as the input data for model calibration has the advantage of including overall and distributed leaf spectral information. It will also reduce the size of the input data for training the model. This method has been widely used for the RGB and HSI imaging (Achanta et al., 2012; Fang et al., 2015).

Overall, the Sp_MLP model showed the best predictive results, followed by Sp_SVM and Sp_PLS-DA. Silva et al. (2013) also suggested that PLS-DA has a lower classification power and is not suitable for weed identification.

The novel approach introduced in this study based on super-pixels (Sp) allows the detection of weeds even where only few parts of the plant are visible, for instance in pastures where these weeds are mixed with other plant species. Thus, the introduced approach helps to overcome the challenging situation where the incomplete visibility of plant’s morphology is a limiting factor for RGB imaging. Also, it is worth noting that spectral signatures could be obtained with non-imaging approaches, but this would have practical challenges in large grazing fields. The HSI used for detection of weeds in our study is either based on unique spectral signature and/or morphological features extracted from the hyperspectral images.

## Conclusion

This study demonstrated the ability of HSI to detect unique spectral signatures of a diverse group of weed species including grass and broadleaf as well as annual and perennial weeds. Models developed with Sp spectral data can provide better results in comparison to averaged spectral data for weed classification. Compared to the traditional classification methods, MLP is a more robust and reliable method when developed with Sp data. This novel approach based on Sp will significantly advance the applicability of HSI in plant identification. This is especially useful when it is applied in the grazing field including in mixed swards of a few plant species. Future work should focus on the development of a system that provides classification using spectral signature and/or morphological features aligned with decision tree strategies to deal with complex systems such as mixed swards.

## Data Availability Statement

Data for this manuscript is available upon request from the corresponding author.

## Author Contributions

KG and MR conceived and planned the experiments. MR and DH carried out the experiments and contributed to sample preparation. YL, MA-S, KI, MR, and KG contributed to the interpretation of the results. YL processed the experimental data and wrote the manuscript with input from all authors. MA-S, KI, GB, MR, and KG contributed to the final version of the manuscript. KG supervised the project. All authors provided critical feedback and helped shape the research.

## Conflict of Interest

MA-S, DH, GB, MR, and KG were employed by the company AgResearch Ltd. KI was employed by the company Red Fern Solutions Ltd. The remaining author declares that the research was conducted in the absence of any commercial or financial relationships that could be construed as a potential conflict of interest.
